# Endolithic Bacterial Diversity in Lichen-Dominated Communities Is Shaped by Sun Exposure in McMurdo Dry Valleys, Antarctica

**DOI:** 10.1007/s00248-021-01769-w

**Published:** 2021-06-03

**Authors:** Ambra Mezzasoma, Claudia Coleine, Ciro Sannino, Laura Selbmann

**Affiliations:** 1 Department of Agricultural, Food and Environmental Sciences, University of Perugia, Borgo XX Giugno 74, 06121 Perugia, Italy; 2 Department of Ecological and Biological Sciences, University of Tuscia, Viterbo, Italy; 3 Italian Antarctic National Museum (MNA), Mycological Section, Genoa, Italy

**Keywords:** Antarctic cryptoendolithic communities, Bacteria, Southern Victoria Land, Sun exposure, Extreme environments

## Abstract

The diversity and composition of endolithic bacterial diversity of several locations in McMurdo Dry Valleys (Continental Antarctica) were explored using amplicon sequencing, targeting the V3 and V4 of the 16S region. Despite the increasing interest in edaphic factors that drive bacterial community composition in Antarctic rocky communities, few researchers focused attention on the direct effects of sun exposure on bacterial diversity; we herein reported significant differences in the northern and southern communities. The analysis of β-diversity showed significant differences among sampled localities. For instance, the most abundant genera found in the north-exposed rocks were *Rhodococcus* and *Blastococcus* in Knobhead Mt.; *Ktedonobacter* and Cyanobacteria Family I *Group I* in Finger Mt.; *Rhodococcus* and *Endobacter* in University Valley; and *Segetibacter* and *Tetrasphaera* in Siegfried Peak samples. In south-exposed rocks, instead, the most abundant genera were *Escherichia/Shigella* and *Streptococcus* in Knobhead Mt.; *Ktedonobacter* and *Rhodococcus* in Finger Mt.; *Ktedonobacter* and *Roseomonas* in University Valley; and *Blastocatella*, Cyanobacteria Family I *Group I* and *Segetibacter* in Siegfried Peak. Significant biomarkers, detected by the Linear discriminant analysis Effect Size, were also found among north- and south-exposed communities. Besides, the large number of positive significant co-occurrences may suggest a crucial role of positive associations over competitions under the harsher conditions where these rock-inhabiting microorganisms spread. Although the effect of geographic distances in these extreme environments play a significant role in shaping biodiversity, the study of an edaphic factor, such as solar exposure, adds an important contribution to the mosaic of microbial biodiversity of Antarctic bacterial cryptoendolithic communities.

**Supplementary Information:**

The online version contains supplementary material available at 10.1007/s00248-021-01769-w.

## Introduction

The McMurdo Dry Valleys (MDV; Southern Victoria Land, Continental Antarctica) represents one of the most remote, driest and coldest environments on Earth. This region is the largest ice-free area in Antarctica [1], characterised by low temperatures, limited snowfalls, high UV radiation and strong katabatic winds. The icy coverage is limited to glaciers and ice-covered lakes, while rocky ice-free mountains and desiccate valleys dominate the landscape. There, the naked rocks supply the main habitat for microbial life [2], hosting the highest standing biomass in this area [3, 4]. Indeed, environmental conditions are so harsh as to be almost incompatible with active life, and microbes can mainly find a refuge dwelling inside rock porosity, forming the so-called endolithic communities, which represent the main and often the sole life-forms in these areas [5].

In these habitats, temperature fluctuations and the thermal inertia of rocks may only occasionally create a transient wetting layer. However, the micro-architectural structure of porous rocks acts as a sponge, prolonging water availability within rock microhabitats which may support the metabolic activity of the endolithic communities in the narrow seasonal window (below 1000 h per year) [6]. Among the different types of endolithic colonisation, the most widespread and known are the cryptoendolithic communities dominated by lichens, colonising quarzitic sandstone as a preferential substratum for its homogeneous porosity and high translucence [7]. These communities are typically organised in a coloured, and biologically distinct, stratification in a depth of about 10 mm below rock surface, according to the physiological requirements of each microbial compartment. Non-lichenised Chlorophyte algae and Cyanobacteria occur in the deepest green band, where harmful solar radiation is more efficiently screened; immediately above, in the white band, lichenised fungi, in obligate symbiosis with algae act, together with phototrophs in the green band, as primary producers, while other fungi and bacteria play a role as consumers [8, 9].

A notable deal of efforts has been recently paid to untangle which environmental (and biological) factors that can putatively drive the composition of Antarctic cryptoendolithic communities or whether environmental factors can diversify highly specialised microbial communities in soils and frozen lakes in the same area of Antarctica [10, 11]. Lee et al. [12] suggested that latitude, pH, nitrate and sulphate concentrations are significantly correlated with the dominant phyla found in rock-inhabiting bacterial communities of several locations in Victoria Land and that rock-inhabiting bacterial communities predominantly consisted of either Actinobacteria or Proteobacteria. Recently, sun exposure was proved to affect Antarctic cryptoendolithic fungal diversity; indeed, more direct sun-exposed rock surfaces (northern exposed rocks) can maintain heating for much longer time due to the thermal inertia of rocks, while microbes may take advantage in terms of water availability (due to ice or snow melting). Both higher temperature and water availability promote metabolic activities with a consequent effect on biodiversity [5, 13, 14]. However, the effect of sun exposure as an environmental factor shaping local community structure and co-occurrence patterns has still never been investigated in Bacteria.

In this work, we aimed (i) to assess rock-inhabiting bacterial communities in the Dry Valleys; (ii) identify marker taxa and estimate the co-occurrences among taxonomicallyrelated bacterial taxa; and (iii) to clarify whether the different exposure (i.e. north vs. south) shapes cryptoendolithic bacterial communities.

## Methods

### Study Area

**Fig. 1:**
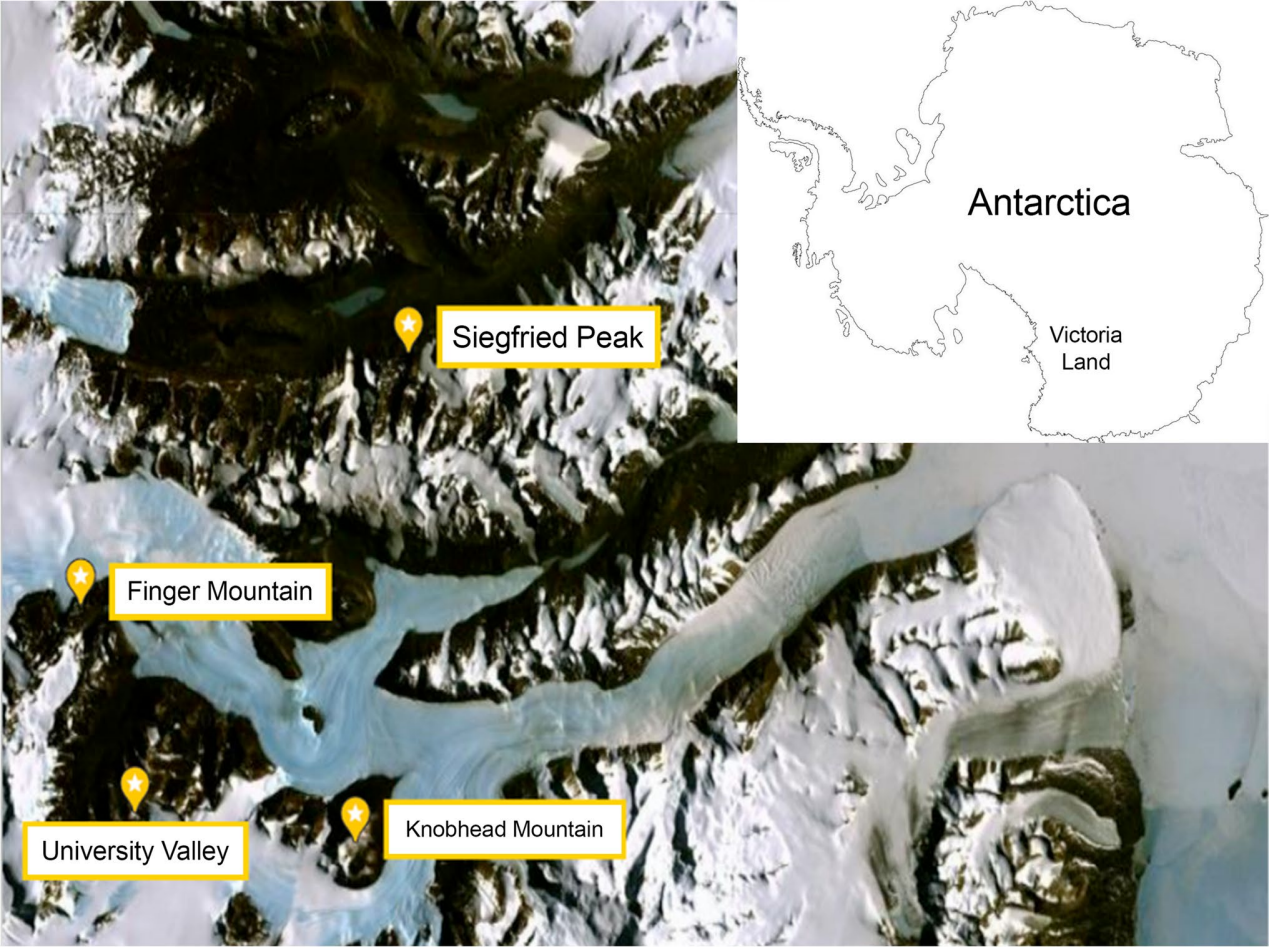
Map of the study area showing the location of sampling sites

The area of study was in MDV, Southern Victoria Land (Continental Antarctica), an ice-free area where the landscape is characterised by huge sandstone outcrop formations of the Beacon Supergroup, of the same geological origin, dating back to the Devonian-Triassic (400 to 250 MYA), with quite homogeneous chemical composition and texture, constituted mostly of orthoquartzite [15, 16]. Sandstone samples were collected during the XXXI Italian Antarctic Expedition (Dec. 2015–Jan. 2016) from four localities (Table 1; Fig. 1; and Supplementary Fig. 1)): (i) Siegfried Peak (SP, number of samples, *n* = 12) is located at the east side of Odin Valley in the Asgard Range of Victoria Land; (ii) Finger Mountain (FM, *n* = 11) is located on the north side of Turnabout Valley, in the group of Quartermain Mountains; (iii) University Valley (UV, *n* = 9) is one of four small valleys in the Quartermain Mountains); (iv) Knobhead (KM, *n* = 8) is an ice-free mountain, standing south of the western end of Kukri Hills and overlooking the Ferrar and Taylor Glaciers. All these sites were located in the Stable Upland Zone (SUZ) microclimatic area, in which the harshest climatic conditions for microbial life occur [17].

**Table 1:** Characteristics of sampling sites: altitude, relative humidity (measured during the sampling), pH and geographic coordinates (modified by Coleine et al., 2020 [13])

Site	Altitude (m a.s.l.)	Relative humidity (%)	pH	Coordinates
Finger Mt. North	1720	35	5.58 ± 0.01	77° 45′ 0.9″ S, 160° 44′ 44.5″ E
Finger Mt. South	1720	35	5.54 ± 0.01	77° 45′ 10.3″ S, 160° 44′ 40.3″ E
Knobhead North	2150	50	4.83 ± 0.01	77° 54′ 37.8″ S, 161° 34′ 48.8″ E
Knobhead South	2150	38	4.76 ± 0.02	77° 54′ 43.6″ S, 161° 34′ 39.3″ E
Siegfried peak North	1620	52	6.11 ± 0.03	77° 34′ 43.3″ S, 161° 47′ 11.7″ E
Siegfried peak South	1620	54	6.22 ± 0.03	77° 34′ 39.9″ S, 161° 47′ 17.4″ E
University Valley North	2090	18	4.57 ± 0.00	77° 52′ 27.6″ S, 160° 44′ 38.9″ E
University Valley North	2200	39	4.69 ± 0.05	77° 52′ 21.6″ S, 160° 45′ 20.5″ E

Both north- and south-exposed rocky surfaces have been collected to highlight the effect of opposite sun exposure on the bacterial assemblages. Conditions for northern-exposed surfaces are, in fact, considerably more favourable than southern-facing rocks that are much cooler, being almost permanently in the shadow [18, 19].

The presence of lithic colonisation was first assessed by direct observation in situ using a magnifying lens. Rock samples, colonised by endolithic lichen-dominated microbial communities, have been collected using a geological hammer and sterile chisel and placed in sterile bags, and then transported and stored at − 20 °C in the Italian National Antarctic Museum—Culture Collection of Fungi from Extreme Environments (MNA-CCFEE). An aliquot of each sample has been shipped to the University of Perugia, Italy, for microbiological analysis. Another aliquot of each sample was used to determine pH (pH meter Hanna Instruments) using oven-dried rock samples and a rock:water ratio of 1:1 [20].

### DNA Extraction and Metabarcoding Sequencing

Total DNA was extracted from 0.25 g of crushed rocks using Power Soil DNA Isolation Kit (Qiagen, Germany) following the manufacturer’s instructions and then quantified using QuBit 2.0 Fluorometer Assay (Life Technologies Corporation). The V3 and V4 regions of the 16S rRNA gene were amplified using the barcoded primers Pro341F: 5′-CCTACGGGNBGCASCAG-3′ and Pro805R: Rev 5′-GACTACNVGGGTATCTAATCC-3′ [21]. PCR solutions and thermocycling conditions were carried out as reported in Coleine et al. [13]. PCR products were then barcoded and pooled. Amplicon sequencing was performed on the Illumina MiSeq platform (2 × 300 bp reads) by BMR Genomics (Padua, Italy).

The 16S dataset was processed by AMPtk (Amplicon ToolKit for NGS) v 1.0.0. [22]. Briefly, reads were demultiplexed and trimmed, and chimeras were removed. Low-quality reads (dropping reads less than 100 bp) and those shorter than 250 bp have been removed and excluded by downstream analysis. The 16S sequences were clustered with VSEARCH v 2.3.2 algorithm [23] using a 97% percent identity parameter to generate the Operational Taxonomic Units (OTUs). Singletons and rare OTUs (< 5 reads across the whole dataset) were additionally removed. Taxonomy was assigned using the hybrid SINTAX/UTAX database [24]. Sequences were submitted to the European Nucleotide Archive (EMBL – EBI) under accession number PRJEB39480.

### Downstream analysis

Shared OTUs and unique OTUs among north and south exposure in each locality were visualised by Venn diagrams. Venn diagrams were plotted using the R packages ‘gplots’, ‘VennDiagram’ and ‘eulerr’ [25–27]. Stacked bar plots at the phylum and genus ranks were obtained using relative abundance. Considering the genus rank, all taxa, which showed relative abundance lower than 2%, were clustered into a taxonomic group named ‘Others’. For each north- and south-exposed surface, alpha diversity metrics, species richness (S), Shannon’s index (H’) and Simpson’s index of dominance (1-D), were calculated. Pairwise comparisons of biodiversity indices among north- and south-exposed rocks were performed by Student’s test, after ANOVA analysis (*p* < 0.05). To explore diversity among localities, beta-diversity analysis was performed using a Bray–Curtis distance matrix and the results were visualised by PCoA plot. The effect of sampling site (locality) on the bacterial community structure was assessed by conducting a permutational ANOVA (PERMANOVA, number of permutations = 9999) with the function ‘adonis’ implemented in the vegan package v. 2.5.5 [28]. Moreover, after transforming the OTU table (square root transformation), Bray–Curtis distance matrices were plotted to show ordinal distances among northern and southern samples in each sampling site with a non-metric multidimensional scaling (NMDS) analysis.

Linear discriminant analysis Effect Size (LEfSe) algorithm (LDA score ≥ 2; *p* value < 0.05; strategy for multi-class analysis ‘all-against-all’) has been implemented to identify the biomarkers (bacterial taxa) associated with each sun exposure, using the Galaxy web interface (https://huttenhower.sph.harvard.edu/galaxy/) [29].

Heatmaps reporting co-occurrences among bacterial phyla were implemented using the Pearson correlation coefficient (*p* < 0.05). R packages ‘lsr’, ‘Hmisc’ and ‘corrplot’ were used for computing correlation matrix and graphical display [30].

## Results

### Characterisation of Bacterial Diversity

After removing OTUs assigned to chloroplasts and Archaea, a total of 893,608 reads have been clustered in 560 OTUs. Venn diagrams reported that all localities shared more than 50% of OTUs, except for samples collected from KM that showed the highest number of unique OTUs in the northern-exposed surface (Supplementary Fig. 2).

**Fig. 2:**
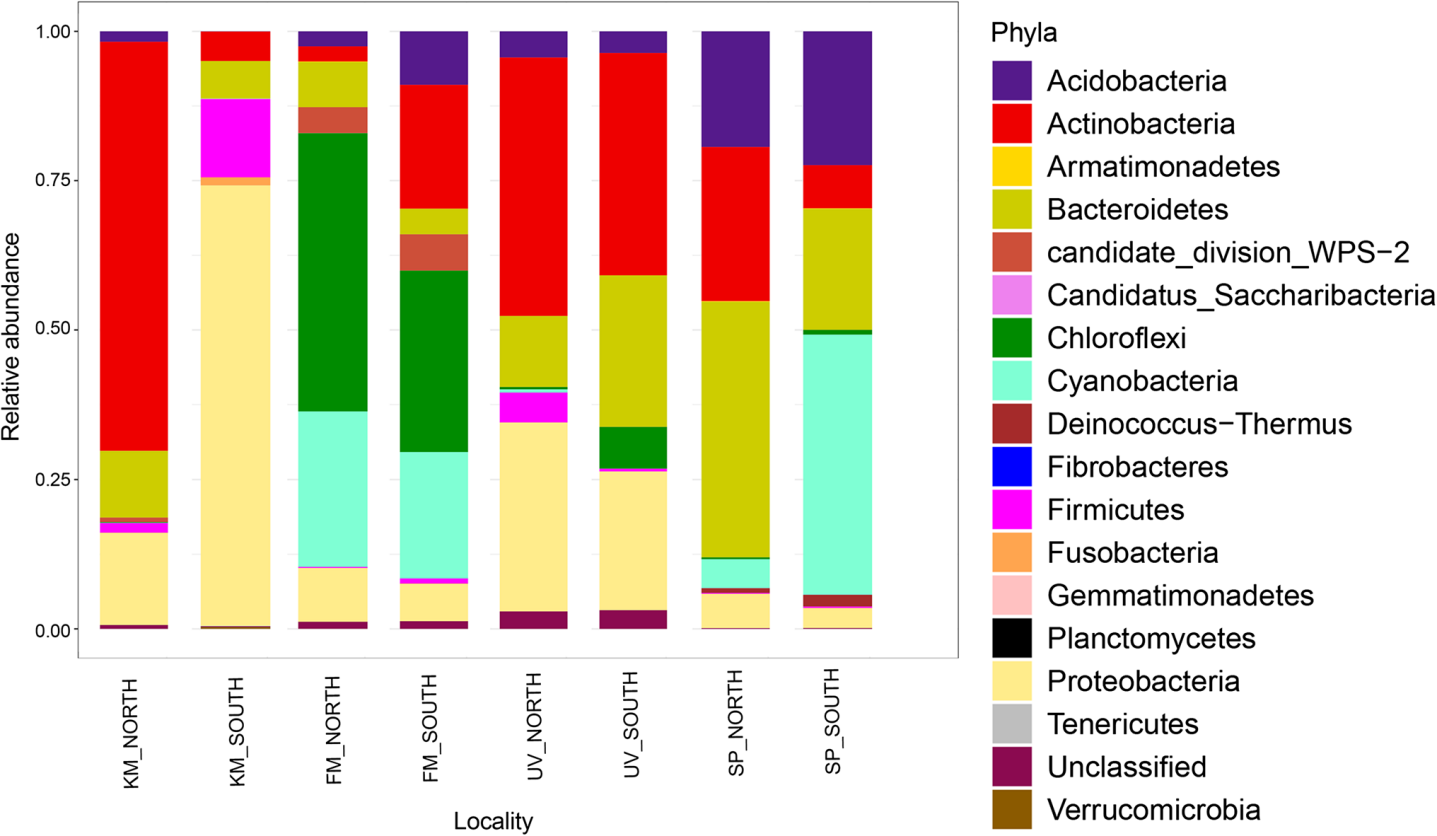
Relative abundances of the dominant bacterial phyla in the cryptoendolithic communities in Victoria Land, Antarctica

The most abundant phyla found in north-exposed rocks were Actinobacteria (68.44, 43.28%) and Proteobacteria (15.42, 31.56%) in KM and UV, respectively, Chloroflexi (46.6%) and Cyanobacteria (25.97%) in FM and Actinobacteria (25.82%) and Bacteroides (42.71%) in SP. On the other hand, in the south-exposed ones, Proteobacteria (73.7%) and Firmicutes (13.09%) were the most abundant phyla in KM, Chloroflexi (30.32%) and Actinobacteria (20.72%) in FM, Actinobacteria (37.23%), Bacteroides (25.37%) and Proteobacteria (23.2%) in UV, Cyanobacteria (43.51%) and Acidobacteria (22.4%) in SP (Fig. 2).

**Fig. 3:**
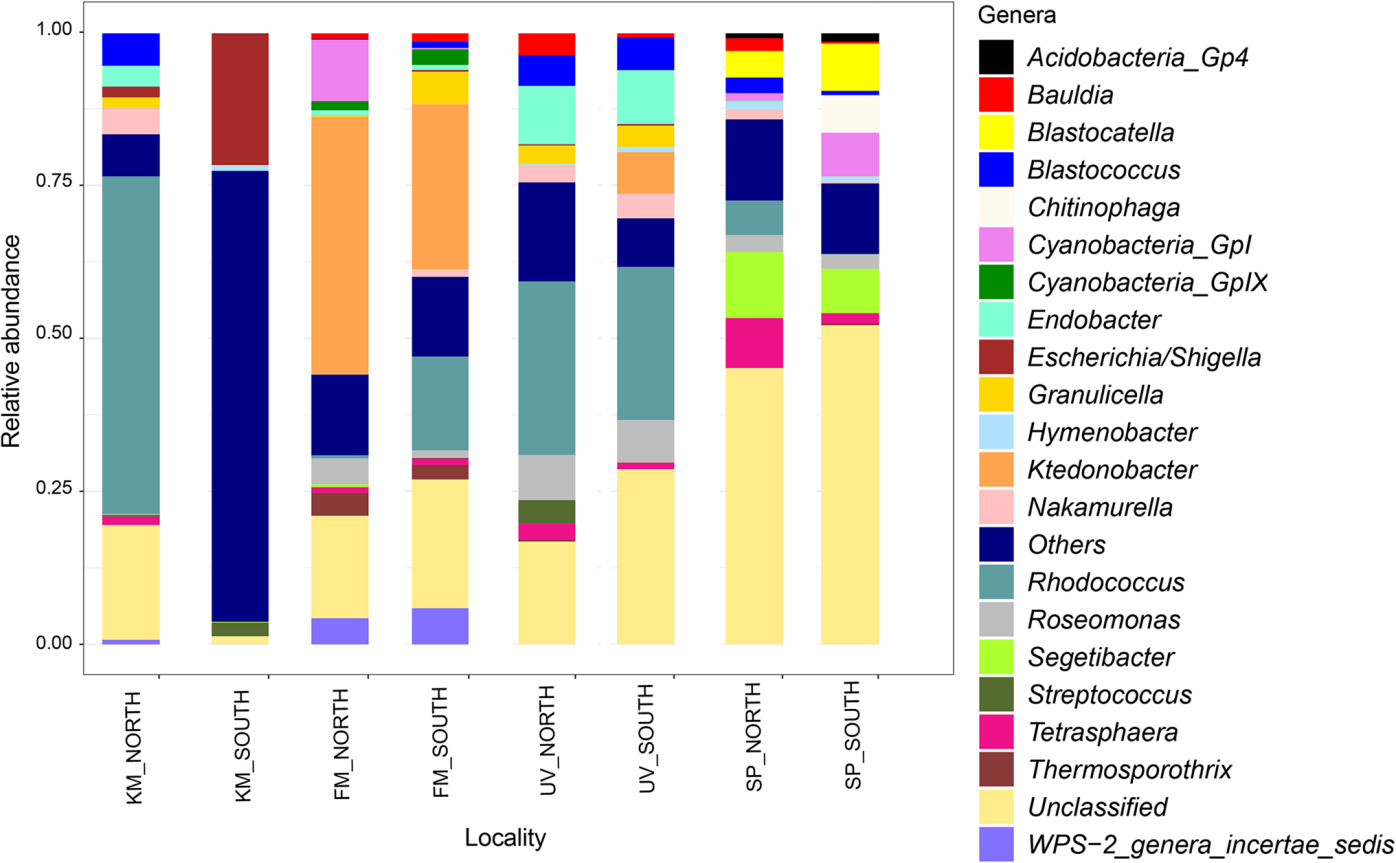
Relative abundances of the dominant bacterial genera (abundance > 1% in two localities) in the cryptoendolithic communities in Victoria Land, Antarctica

More than 200 genera were found in the examined rocks. *Blastocatella*, *Blastococcus*, *Ktedonobacter*, *Roseomonas*, *Rhodococcus*, *Segetibacter* and *Tetrasphaera* showed a relative abundance higher than 1% at least in two samples. Unclassified OTUs at the genus level were found in all sites, and their abundance amounts to 34% of the total. Overall, the south-exposed samples reported a higher number of unclassified OTUs at genus level than the north ones (Fig. 3).

The most abundant genera found in north-exposed rocks were *Rhodococcus* (56.3%) and *Blastococcus* (5.54%) in KM, *Ktedonobacter* (42.75%) and Cyanobacteria Family I *GpI* (10.2%) in FM, *Rhodococcus* (29.6%) and *Endobacter* (9.96%) in UV and *Segetibacter* (11.03%) and *Tetrasphaera* (8.33%) in SP sample. Regarding south-exposed rocks, the most abundant genera were as follows: *Escherichia*/*Shigella* (48.68%) and *Streptococcus* (2.96%) in KM, Ktedonobacter (27.83%) and Rhodococcus (15.80%) in FM, *Ktedonobacter* (25.30%) and *Roseomonas* (7%) in UV and *Blastocatella* (7.9%), Cyanobacteria Family I *GpI* (7.31%) and *Segetibacter* (7.42%) in SP.

### Effects of Sun Exposure on Biodiversity and Community Composition

Considering the different exposure in the studied localities, only SP showed a significant difference (*p* < 0.05) between north- and south-exposed rocks in species richness (Supplementary Table 1). Shannon’s index (H’) and Simpson index (1-D) did not show any significant differences (Student’s Test, *p* > 0.05) between north- and south-exposed samples in each locality.

**Fig. 4:**
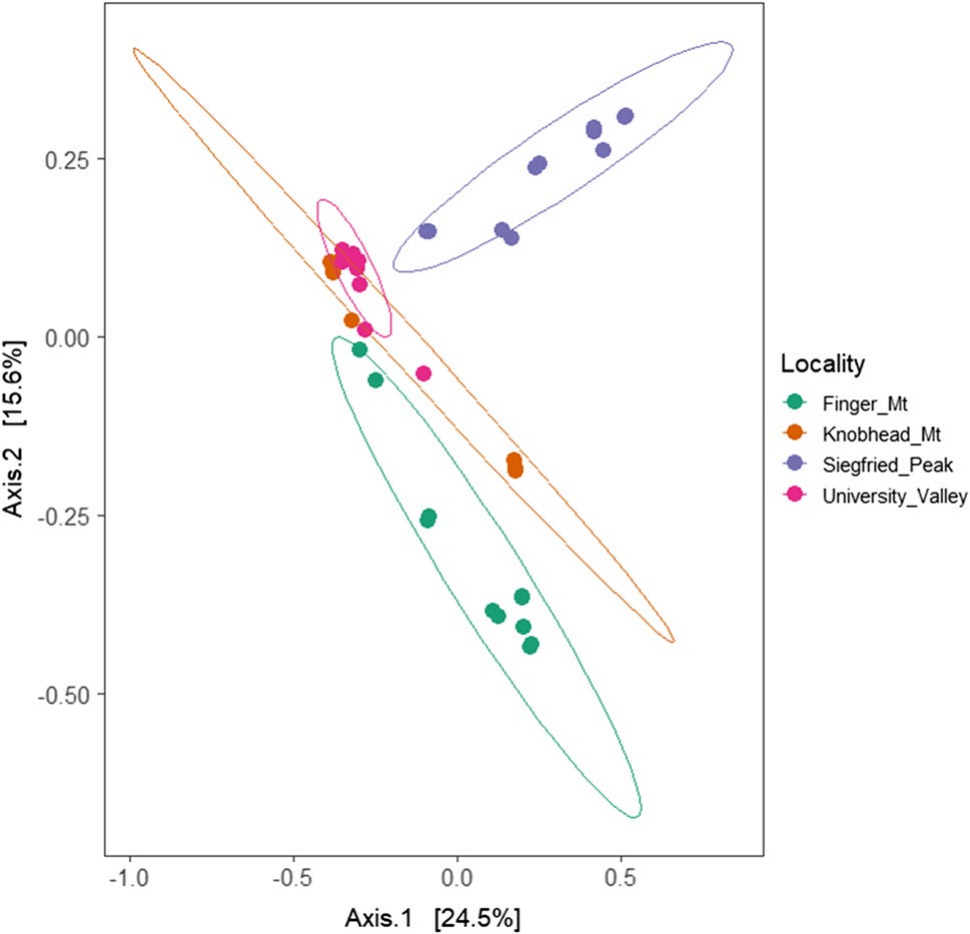
Principal coordinates analysis based on Bray–Curtis distance matrix and calculated using all bacterial OTUs. The effect of sampling site (locality) on bacterial community structure was assessed by conducting a permutational ANOVA (PERMANOVA, number of permutations = 9,999). *p* value = 0.001

**Fig. 5:**
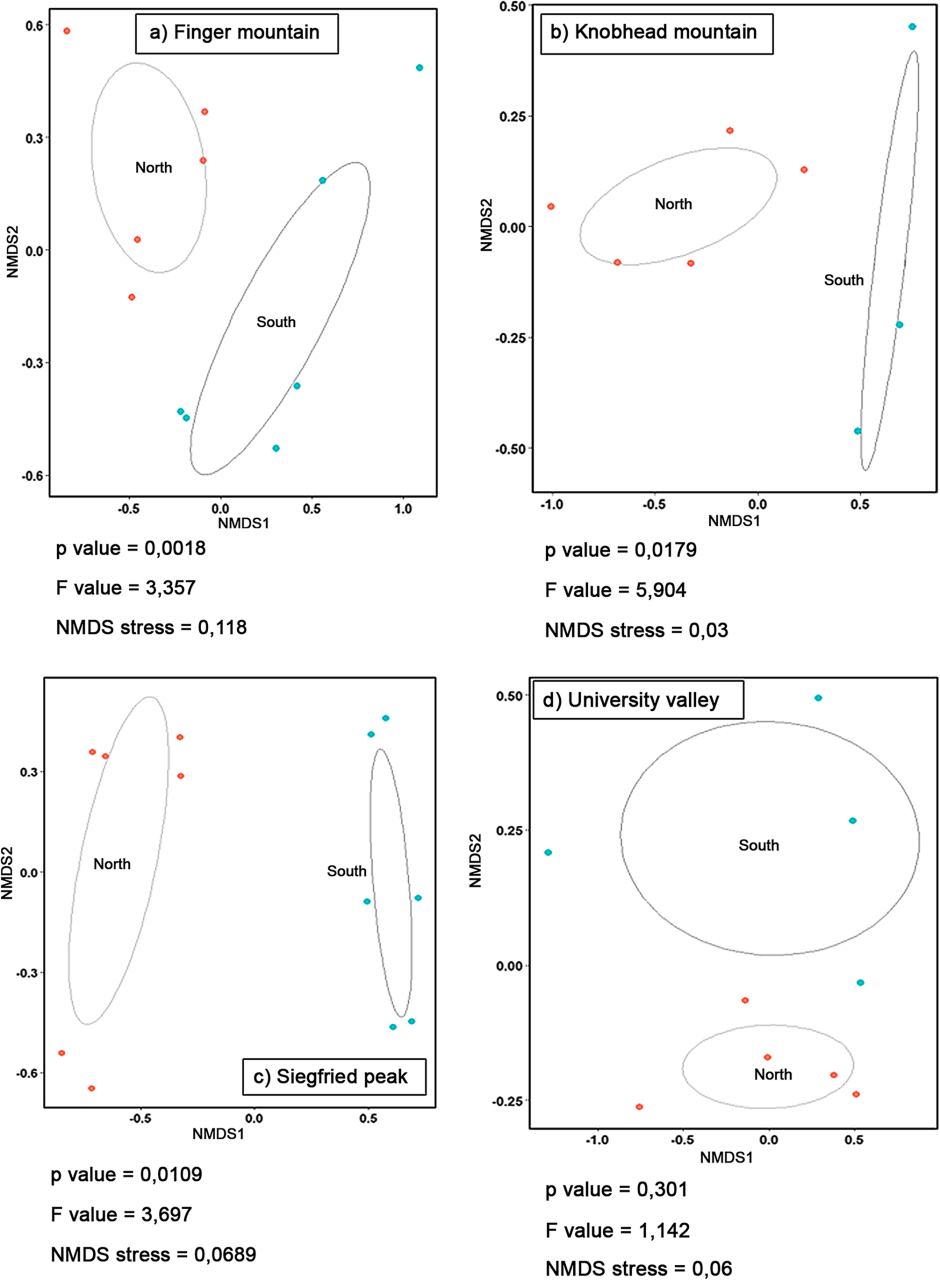
Non-metric NMDS and PERMANOVA analysis of sequences from North- and South-facing rocks, based on square root-transformed abundance data. Compositional dissimilarity was calculated using Bray–Curtis distance, and ellipses plotted using multivariate normal distribution at a confidence level of 95%

Dissimilarity in community composition showed significant differences (*p* < 0.05) among the four localities studied (Fig. 4). Likewise, north- and south-exposed rocks showed significant differences in community composition in FM (*p* = 0.0018), in KM (*p* = 0.0179) and in SP (*p* = 0.0109). No significant differences (*p* = 0.301) were detected for UV samples (Fig. 5).

The LEfSe analysis showed differences among differently exposed rocks in FM, as follows: *Ktedonobacter*, Cyanobacteria Family I *Group I*, *Sphingobacterium*, *Roseomonas* and *Mucilaginibacter* at the genus level and Acetobacteraceae and Chitinophagaceae at family level for north-exposed rocks; instead, in south-exposed rocks of FM, the relevant biomarkers belonged to *Rhodococcus*, *Ktedonobacter*, *Granulicella* and *Nakamurella* genera, Nocardiaceae and Enterobacteriaceae at the family level, to Actinobacteria at phylum and class level. In north-exposed rocks of SP, the relevant taxa were Actinobacteria at the phylum level, Nocardiaceae, Intrasporangiaceae and Nakamurellaceae at family rank and *Tetrasphaera*, *Rhodococcus* and *Nakamurella* for genus level; on the contrary, Cyanobacteria were the most important biomarker in SP south-exposed samples. Bacteroidetes phylum and its class, Sphingobacteria, together with Alphaproteobacteria class, showed the highest LDA score in north-exposed samples collected in KN; differently, the classes Bacilli, Gammaproteobacteria and Betaproteobacteria, and the genus *Propionibacterium* were the discriminating taxa in southern surfaces. Finally, at UV, the north- and south-exposed rocks differ at genus level: *Endobacter* and *Ehrlichia* characterised the north exposure, whereas *Desulfotomaculum* and *Acidovorax* were present in the south (Supplementary Figs. 3, 4 and 5).

**Fig. 6:**
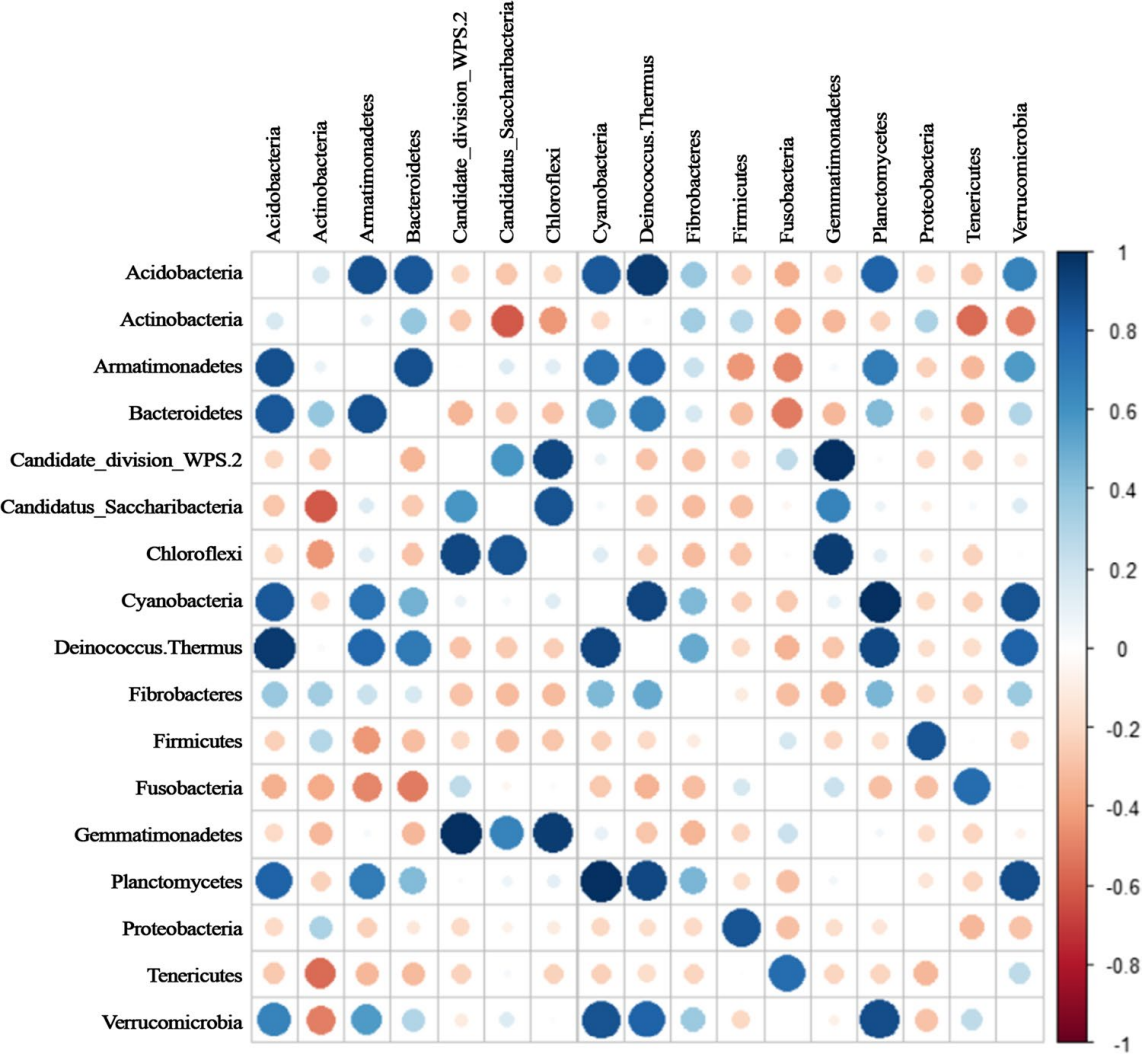
Heatmap summarising pairwise significant (*p* < 0.05) correlations among bacterial OTUs occurring in ice-free rocks under study (McMurdo Dry Valleys, Antarctica). The color key for the correlation values is shown on the right of the figure. The analysis was performed considering the OTUs classified at phylum level by Pearson coefficient (relative abundance data > 1%)

A total of 21 positive pairwise significant (*p* < 0.05) correlations were found among OTU abundances assigned to phylum level: Gemmatimonadetes vs. candidate division WPS-2 (*r* = 0.99); Planctomycetes vs. Cyanobacteria (*r* = 0.99) and Deinococcus Thermus vs. Acidobacteria (*r* = 0.95) showed the highest value by Pearson correlation (Fig. 6; Supplementary Table 2).

## Discussion

The effect of sun exposure on bacterial assemblages in the lichen-dominated communities, dwelling inside Antarctic rocks, collected from four sites of MDV (accounted as the closest Martian analogue on Earth) was explored. North- and south-exposed rocks were considered to highlight the effect of the different environmental pressures due to opposite sun exposure. These microorganisms live close to the limit of their tolerance, and the change in sun exposure may critically affect the conditions for life [31].

Actinobacteria was the most abundant phylum and almost all the representative OTUs, belonging to this phylum, were assigned to *Blastococcus* and *Tetrasphaera*. Both genera were rarely found in Antarctica, although Pudasaini et al. [32] hypothesised that the low frequency of isolation of the genus *Blastococcus* and other endemic genera in Antarctica could be related to methodological issues in culture-dependent approach. Crits-Christoph et al. [33] performed metagenomic analyses of bacterial communities from two rock substrates (calcite and ignimbrite) in the Atacama Desert and revealed that Bacteroidetes were represented by *Segetibacter* in the ignimbrite microbial community. This genus occurred in SP and KM south sites, suggesting the peculiar adaptation of this genus to extreme habitats. Finally, the genus *Roseomonas* was the most representative taxon for the phylum Proteobacteria, and the reads assigned to this genus were uniformly distributed over all the analysed locations. In previous studies concerning Antarctic environments, this genus was recovered in Antarctic Dry Valleys soils, in the perennially ice-covered Lake Untersee, and a water column of freshwater Lake Radok [34–37], confirming the cosmopolitan aptitude of this genus.

Species richness exhibited a significant difference (*p* < 0.05) between north- and south-exposed rocks only in Siegfried Peak site. Besides, it might be considered that the south-exposed surfaces in this locality were positioned in a depression of the rock surface creating a protective niche that allowed the development of epilithic lichens to a certain extent (Supplementary Fig. 1h ). The more favourable conditions may have led to the higher diversification found compared not only to north-exposed surfaces but also to all other analysed locations.

Results of beta-diversity reported significant differences among the studied sites. The factor ‘habitat’ (sampled localities) accounted for 35% of the total community variation among samples. Siegfried Peak, located in a more distant position compared to the others, falls apart in the diagram, denoting that biodiversity is related to geographic distance, particularly in these remote and inhospitable environments where the spreading and successful settlement of bacterial cells is a challenge, promoting the evolution of local populations.

North- and south-exposed rocks showed significant differences in community composition (*p* < 0.05), except for University Valley. These data proved that sun exposure significantly influences and shapes the biodiversity of bacterial communities. Besides, the geochemically quite homogeneity of rock between north- and south-exposed samples, the same area of sampling (MDV) and the comparable altitudes [15, 16] can make other environmental factors to be considered negligible.

By LEfSe analysis, the relative abundance of *Rhodococcus* and *Ktedonobacter* genera were significantly different between the north- and south-exposed samples in SP and FM and, therefore, can be considered biomarkers of these two conditions. The genus *Rhodococcus* includes the most common species recorded from Antarctic environments [38, 39]. Members of this genus can grow in a broad range of temperatures, below 0 °C up to 50 °C [40]. The adaption to cold was also demonstrated by Goordial et al. [41] that isolated from Antarctic Dry Valley permafrost, *Rhodococcus* sp. JG-3, capable of growth from 30 down to − 5 °C. Several studies, moreover, focused attention on the abilities of some *Rhodococcus* strains to degrade phenols and hydrocarbons in contaminated soils in Antarctica [42, 43]. The genus *Rhodococcus* and even sequences assigned to the family Nocardiaceae, to which it belongs, were widespread, being found almost in all sites. The uncultured genus *Ktedonobacter* was found with high relative abundance (> 10%) but at FM only. High relative abundances of Ktedonobacteria are commonly found in extreme environments [44, 45]. However, the enormous variety of environmental conditions (high and low temperatures, nutrient-rich and oligotrophic substrates, high and low pH), where Ktedonobacteria class can thrive, suggests that these Gram-positive, aerobic and mycelia-forming actinomycetes-like bacteria could be able to produce secondary metabolites, to survive in extremely different conditions and different geochemical rock surfaces [46, 47]. In all samples, few reads were assigned to *Thermosporothrix*, which belonged to Ktedonobacteria. This finding suggests setting further culturable conditions to isolate new strains in this genus because, to the best of our knowledge, only thermophilic strains can be successfully cultivated [48].

Among the most representative members of these communities, some phyla have already been found in other previous works [9, 49], but focusing attention on sun exposure and different sampling sites, some poorly studied microorganisms and their unexplored interactions emerged. For instance, Gemmatimonadetes are frequently detected in soils and their relative abundance in 16S rRNA gene libraries (> 500 sequences) is from 0.2 to 6.5% [50]. In Antarctica, their relative abundance was even higher in a pyrosequencing investigation (15%) of lithobionts and soil niches from Victoria Valley [51]. Their cosmopolitan distribution in terrestrial environments seems to be related to pH and soil moisture; in particular, the highest relative abundances were observed where pH is neutral and several studies in arid and semiarid habitats reported many Gemmatimonadetes phylotypes, suggesting a possible adaptation to drier soils like those found in the MDV [50]. A positive significant correlation between Gemmatimonadetes and candidate division WPS-2 suggests to deeply investigate their metabolisms and primary energy sources, also in the light of a recent study, where genes, supporting CO_2_ fixation through the Calvin-Benson-Bassham (CBB) cycle, were found in candidate division WPS-2, Actinobacteria and AD3 phyla in Antarctic desert soils [52]. Moreover, the role of temperature should be studied on the growth of candidate division WPS-2 phylum because these two phyla frequently occur in the same habitats with different abundances [44, 53]. Moreover, phototrophic members of candidate division WPS-2 seem to prefer cold, acidic and aerobic environments with access to sunlight [54]. Another interesting correlation is between Cyanobacteria and Planctomycetes. Cyanobacteria are commonly identified in Antarctic endolithic communities, and this phylum is commonly found in hypolithic and chasmoendolithic microbial communities [6, 49, 55, 56]. Cyanobacteria were abundant in two localities (FM and SP) only; their spatial distribution confirms previous studies in which Cyanobacteria abundances vary greatly even across Antarctic soils, mainly depending on water sources [44, 57]. Although reads assigned to Planctomycetes were recovered even in rocks, this phylum is typically found in freshwater samples, marine sediments, cryoconite holes or associated with soft corals in Antarctica, suggesting a more specific adaptation to habitats where liquid, salty water is available [58–60]. However, in these peculiar rock niches, they may take advantages of the following situations that may favour their settlement and success: (i) the presence of Cyanobacteria, among which some taxa are aerobic nitrogen fixing and their metabolic activities could be the source of ammonium, used by some Planctomycetes anammox genera; (ii) the transient presence of liquid water due to freezing/thawing of water in the undersides and the interstices of rocks; and (iii) the high salt concentration that may occur on rock surface due to the strong evaporation of water, resulting in salt efflorescence on rock surfaces [61]. Furthermore, Cai et al. suggested that the abundance of Planctomycetes phylum in cyanobacterial blooms could be related to the degradation of the sulphated polysaccharides produced by Cyanobacteria in the Chinese eutrophic Lake Taihu [62]. Another positive relationship showing strong co-occurrence patterns between Cyanobacteria and Planctomycetes was also found in eutrophic lakes in China [63].

Bacterial community correlations and different phyla distribution in sampling sites could be also explained considering the presence of lichens. Culturing approaches have revealed the presence of Actinobacteria and Proteobacteria as major phyla associated with Antarctic lichens [9, 12, 64]. A recent study, carried out through 454 pyrosequencing, added Acidobacteria and Bacteroidetes phyla to predominant bacterial phyla in Antarctic lichens [65]. Niederberger et al. [36] revealed that bacterial communities grouped in distinct clusters specific to arid and wet soils and both Acidobacteria and Deinococcus-Thermus phyla shared the higher abundances in the arid MDV soils with Bacteroidetes, Firmicutes and Gemmatimonadetes, contrary to Cyanobacteria, which preferred wetted soils. Although representatives of the phylum Deinococcus Thermus have been frequently isolated in Antarctic environmental samples [9, 66], in this study, a very few reads were assigned to this taxon; the significant positive correlation with Acidobacteria is worth to be investigated in the future considering the edaphic features of Siegfried Peak samples where the highest number of reads was retrieved for both these phyla.

This study evidenced that opposite sun exposure may influence bacterial assemblage of the cryptoendolithic lichen-dominated communities in terms of diversity and composition, and for the presence of marker species, even if to a lesser extent compared to fungi [14, 67]. We also found, as previously reported, a high local diversification more pronounced along with longer distances [2, 14, 68, 69]. Apparently, the effect of geographic distances in these remote and inhospitable environments plays a determinant role in shaping biodiversity since the successful settlement of bacterial cells is extremely challenging and may be based on an incidental success as for the founder or bottleneck effects promoting the evolution of local populations. The evidence of positive correlations underlines the importance of strong cooperation to withstand the border conditions of the studied rocks, rather than competition. Although the biodiversity of prokaryotes in Dry Valleys’ rocks is still far from being fully described, the study of an edaphic factor, such as solar exposure, adds an important contribution to the mosaic of microbial biodiversity of Antarctic cryptoendolithic communities.

## Supplementary Information

Below is the link to the electronic supplementary material.

Supplementary file1 (DOCX 2783 KB)

## Data Availability

The data are available in European Nucleotide Archive (EMBL – EBI) under accession number PRJEB39480.

## Code Availability

Not applicable for that section.
