# Supplementary material for: Endolithic Bacterial Diversity in Lichen-Dominated Communities Is Shaped by Sun Exposure in McMurdo Dry Valleys, Antarctica
Source: Microb Ecol. 2021 Jun 3;83(2):328–39. doi: 10.1007/s00248-021-01769-w (PMC8891110; doi:10.1007/s00248-021-01769-w)

**Endolithic bacterial diversity in lichen-dominated communities is shaped by sun exposure in McMurdo Dry Valleys, Antarctica.**

**Ambra Mezzasoma, Claudia Coleine, Ciro Sannino and** **Laura Selbmann.**

**Supplementary Fig.1** Map of sampling sites. 1a) Finger mountain sampling area north exposure; 1b) Finger mountain sampling area south exposure; 1c) University valley sampling area north exposure; 1d) University valley sampling area south exposure; 1e) Knobhead mountain sampling area north exposure; 1f) Knobhead mountain sampling area south exposure; 1g) Siegfried peak sampling area north exposure; 1h) Siegfried peak sampling area south exposure and outcrop.


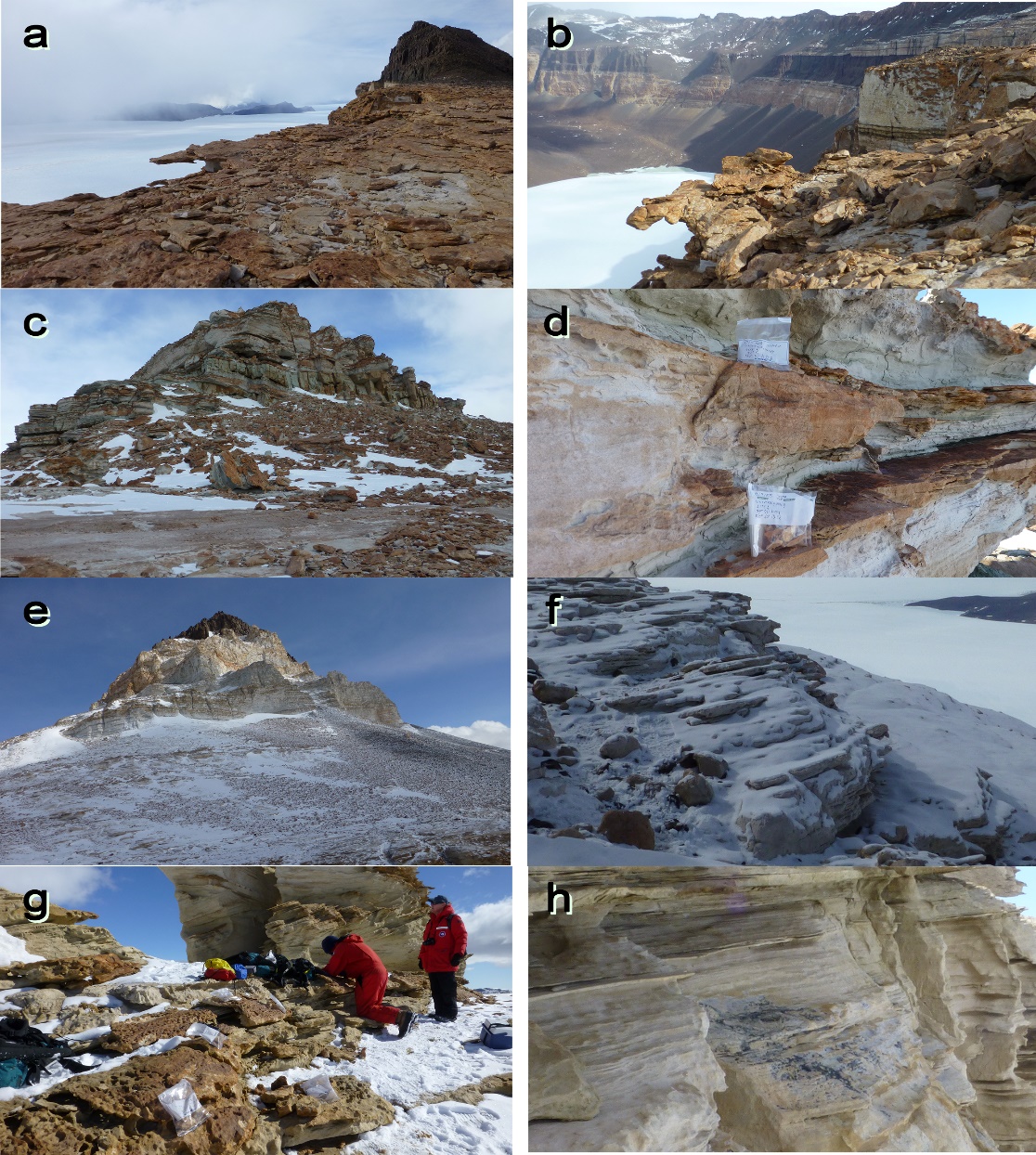


**Supplementary Fig.2** Venn diagrams showing the numbers of bacterial OTUs shared between north and south exposed rocks in each locality.


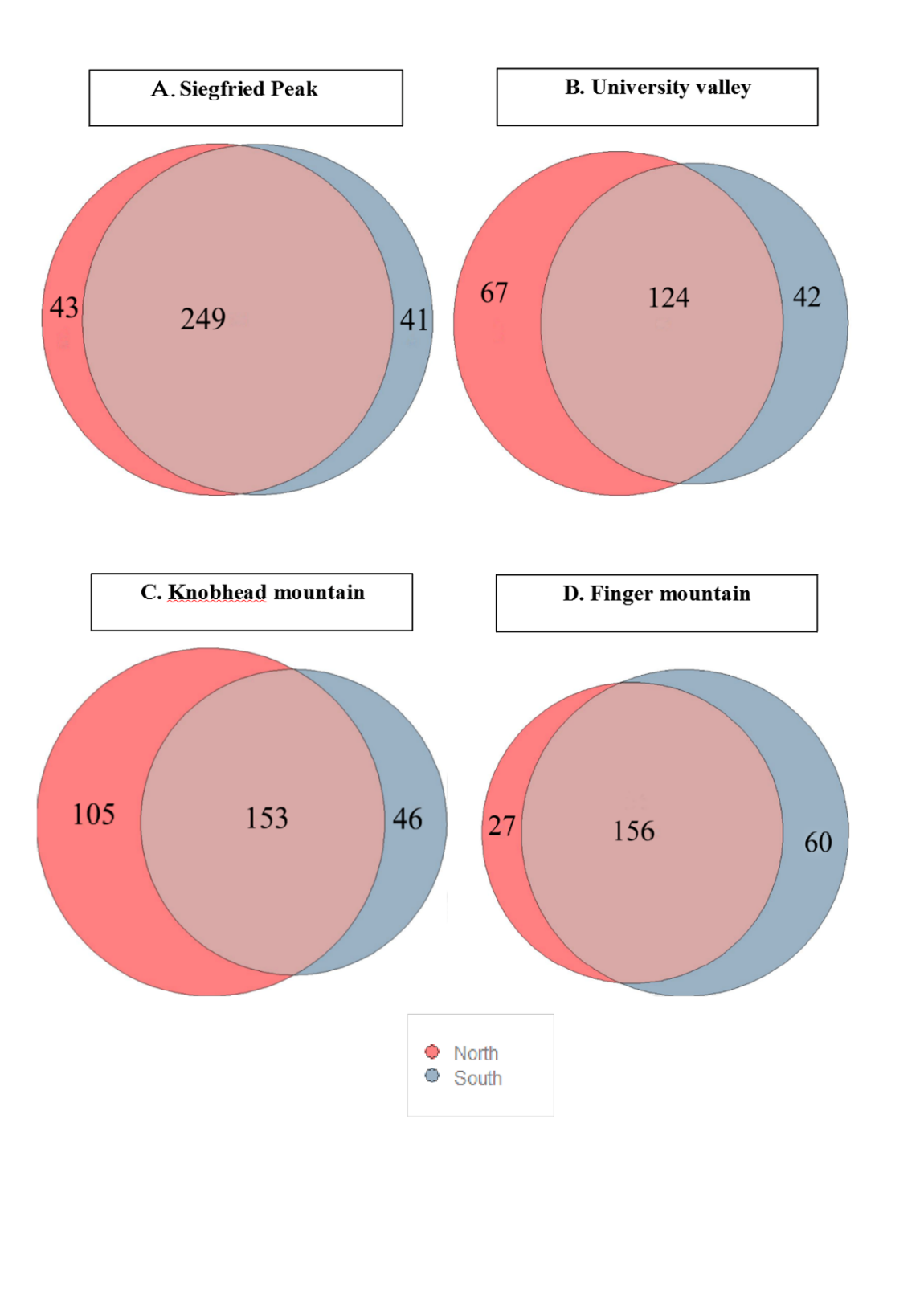


**Supplementary Fig. 3** Linear discriminant analysis Effect Size (LEfSe) algorithm (LDA score ≥ 2; p-value < 0.05; strategy for multi-class analysis “all-against-all”) on OTUs recovered in Finger mountain site.


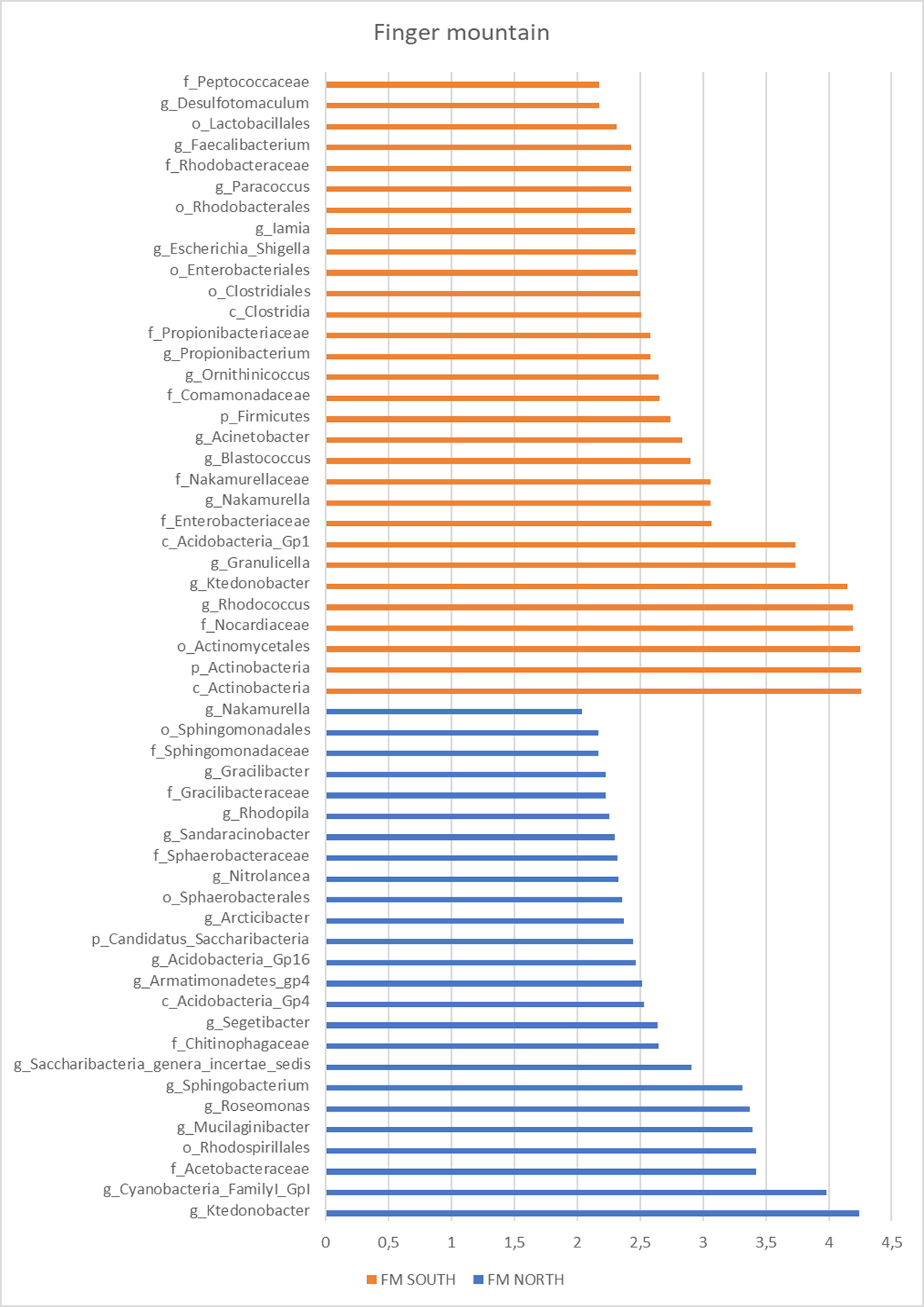


**Supplementary Fig. 4** Linear discriminant analysis Effect Size (LEfSe) algorithm (LDA score ≥ 2; p-value < 0.05; strategy for multi-class analysis “all-against-all”) on OTUs recovered in Siegfried peak site.


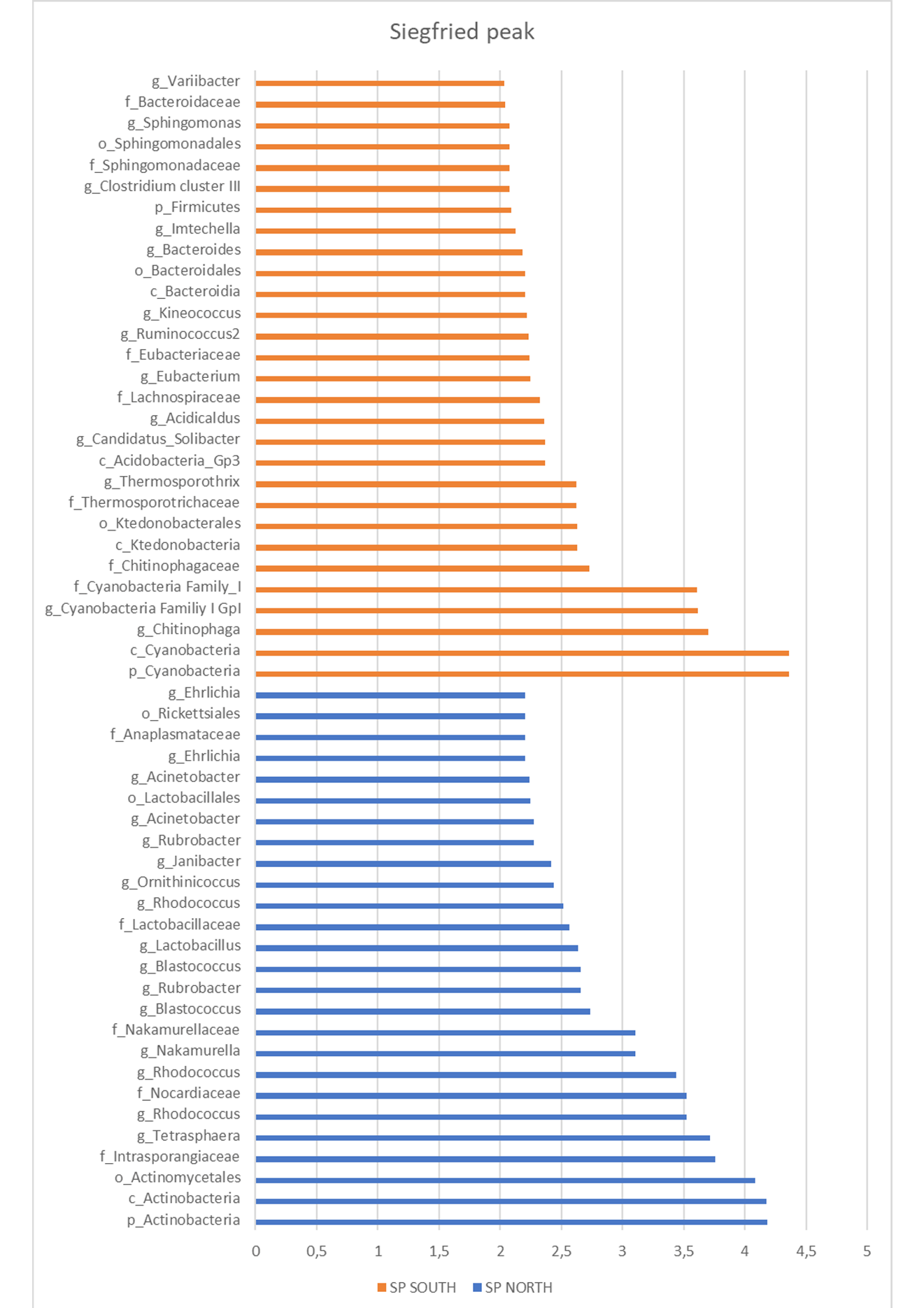


**Supplementary Fig. 5** Linear discriminant analysis Effect Size (LEfSe) algorithm (LDA score ≥ 2; p-value < 0.05; strategy for multi-class analysis “all-against-all”) on OTUs recovered in Knobhead mountain site and University valley site.


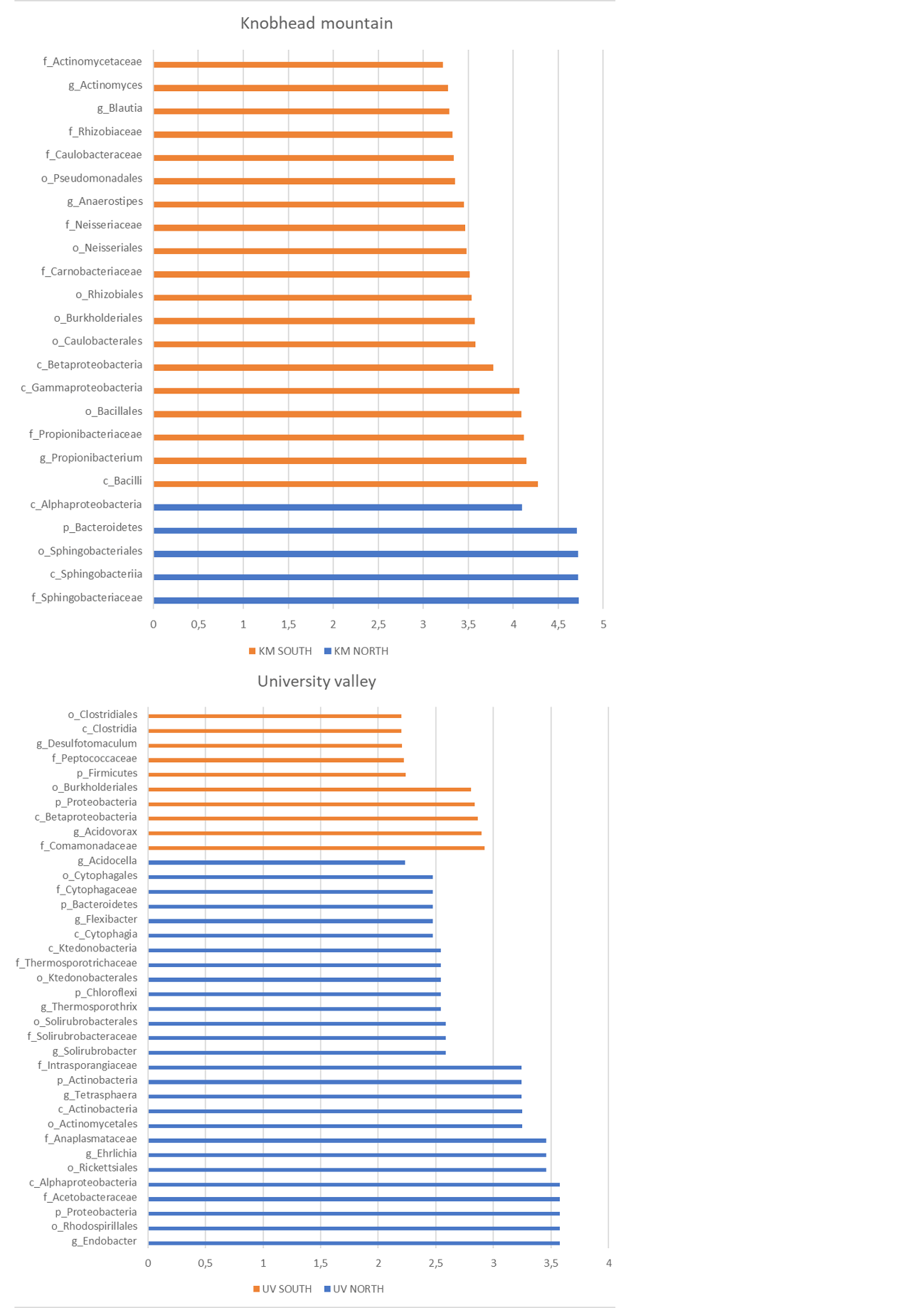


**Supplementary Table 1** Alpha diversity (Species Richness, Shannon and Simpson diversity indices) of sampling localities, considering north and south exposed rocks. The t-values were calculated by Student's t-test. Localities sharing the same letter are not significantly different.


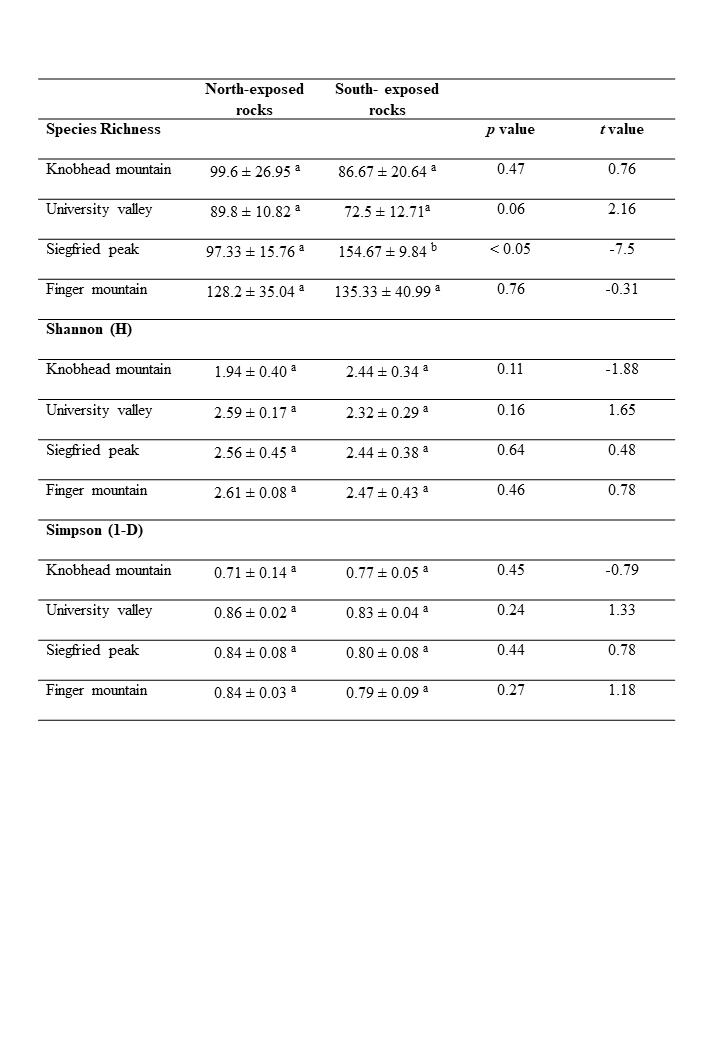


**Supplementary Table 2.** Significant correlations (p < 0.05) among bacterial OTUs assigned to phylum level and analyzed by Pearson’s correlation.


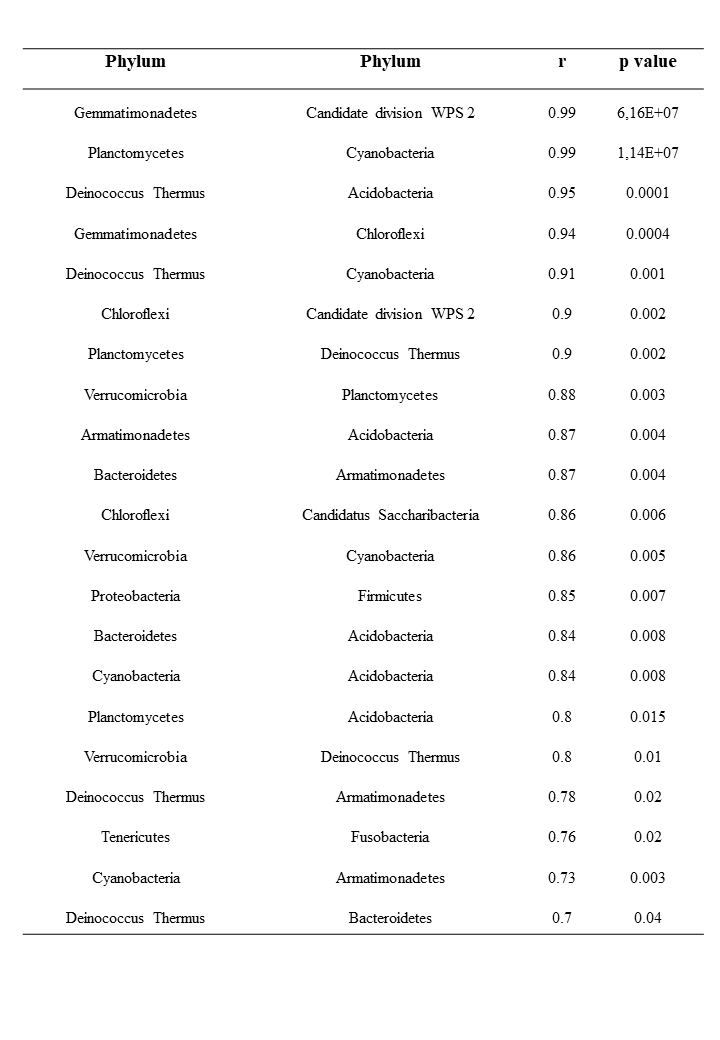

Supplement: Supplementary file 1 — Supplementary file1 (DOCX 2783 KB) [file 248_2021_1769_MOESM1_ESM.docx]
